# High-throughput discovery of plastid genes causing albino phenotypes in ornamental chimeric plants

**DOI:** 10.1093/hr/uhac246

**Published:** 2022-11-03

**Authors:** Hyun-Seung Park, Jae-Hyeon Jeon, Woohyeon Cho, Yeonjeong Lee, Jee Young Park, Jiseok Kim, Young Sang Park, Hyun Jo Koo, Jung Hwa Kang, Taek Joo Lee, Sang Hoon Kim, Jin-Baek Kim, Hae-Yun Kwon, Suk-Hwan Kim, Nam-Chon Paek, Geupil Jang, Jeong-Yong Suh, Tae-Jin Yang

**Affiliations:** Department of Agriculture, Forestry and Bioresources, Research Institute of Agriculture and Life Sciences, and Plant Genomics and Breeding Institute, College of Agriculture and Life Sciences, Seoul National University, Seoul, 08826, Republic of Korea; Department of Integrative Biological Sciences and Industry, Sejong University, Seoul 05006, Korea; Department of Agriculture, Forestry and Bioresources, Research Institute of Agriculture and Life Sciences, and Plant Genomics and Breeding Institute, College of Agriculture and Life Sciences, Seoul National University, Seoul, 08826, Republic of Korea; Department of Agriculture, Forestry and Bioresources, Research Institute of Agriculture and Life Sciences, and Plant Genomics and Breeding Institute, College of Agriculture and Life Sciences, Seoul National University, Seoul, 08826, Republic of Korea; Department of Agriculture, Forestry and Bioresources, Research Institute of Agriculture and Life Sciences, and Plant Genomics and Breeding Institute, College of Agriculture and Life Sciences, Seoul National University, Seoul, 08826, Republic of Korea; Department of Agriculture, Forestry and Bioresources, Research Institute of Agriculture and Life Sciences, and Plant Genomics and Breeding Institute, College of Agriculture and Life Sciences, Seoul National University, Seoul, 08826, Republic of Korea; Department of Agriculture, Forestry and Bioresources, Research Institute of Agriculture and Life Sciences, and Plant Genomics and Breeding Institute, College of Agriculture and Life Sciences, Seoul National University, Seoul, 08826, Republic of Korea; Department of Agriculture, Forestry and Bioresources, Research Institute of Agriculture and Life Sciences, and Plant Genomics and Breeding Institute, College of Agriculture and Life Sciences, Seoul National University, Seoul, 08826, Republic of Korea; Department of Agriculture, Forestry and Bioresources, Research Institute of Agriculture and Life Sciences, and Plant Genomics and Breeding Institute, College of Agriculture and Life Sciences, Seoul National University, Seoul, 08826, Republic of Korea; Hantaek Botanical Garden, Yongin, Gyeonggi-do, 17183, Republic of Korea; Hantaek Botanical Garden, Yongin, Gyeonggi-do, 17183, Republic of Korea; Radiation Breeding Research Team, Advanced Radiation Technology Institute, Korea Atomic Energy Research Institute, Jeongeup 56212, Korea; Radiation Breeding Research Team, Advanced Radiation Technology Institute, Korea Atomic Energy Research Institute, Jeongeup 56212, Korea; Special Forest Resources Division, National Institute of Forest Science, Suwon 16631, Korea; Department of Agriculture, Forestry and Bioresources, Research Institute of Agriculture and Life Sciences, and Plant Genomics and Breeding Institute, College of Agriculture and Life Sciences, Seoul National University, Seoul, 08826, Republic of Korea; Department of Agriculture, Forestry and Bioresources, Research Institute of Agriculture and Life Sciences, and Plant Genomics and Breeding Institute, College of Agriculture and Life Sciences, Seoul National University, Seoul, 08826, Republic of Korea; School of Biological Sciences and Technology, Chonnam National University, Gwangju, South Korea; Department of Agricultural Biotechnology and Research Institute of Agriculture and Life Sciences, Seoul National University, Seoul 08826, Korea; Department of Agriculture, Forestry and Bioresources, Research Institute of Agriculture and Life Sciences, and Plant Genomics and Breeding Institute, College of Agriculture and Life Sciences, Seoul National University, Seoul, 08826, Republic of Korea

## Abstract

Chimeric plants composed of green and albino tissues have great ornamental value. To unveil the functional genes responsible for albino phenotypes in chimeric plants, we inspected the complete plastid genomes (plastomes) in green and albino leaf tissues from 23 ornamental chimeric plants belonging to 20 species, including monocots, dicots, and gymnosperms. In nine chimeric plants, plastomes were identical between green and albino tissues. Meanwhile, another 14 chimeric plants were heteroplasmic, showing a mutation between green and albino tissues. We identified 14 different point mutations in eight functional plastid genes related to plastid-encoded RNA polymerase (rpo) or photosystems which caused albinism in the chimeric plants. Among them, 12 were deleterious mutations in the target genes, in which early termination appeared due to small deletion-mediated frameshift or single nucleotide substitution. Another was single nucleotide substitution in an intron of the *ycf3* and the other was a missense mutation in coding region of the *rpoC2* gene. We inspected chlorophyll structure, protein functional model of the rpoC2, and expression levels of the related genes in green and albino tissues of *Reynoutria japonica*. A single amino acid change, histidine-to-proline substitution, in the rpoC2 protein may destabilize the peripheral helix of plastid-encoded RNA polymerase, impairing the biosynthesis of the photosynthesis system in the albino tissue of *R. japonica* chimera plant.

## Introduction

The global indoor plant market is predicted to reach approximately $7.27 billion by 2025 [1] and to grow more rapidly as a consequence of the increased time spent indoors due to the COVID-19 pandemic. Chimeric plants provide an attractive decoration source in indoor and outdoor settings. Since the description of the first chimera, a graft hybrid between bitter orange (*Citrus aurantium*) and citron (*Citrus medica*) called “Bizzaria”, in 1674 [2], many chimeras arising naturally or artificially have been reported and sold on the market. The mechanisms by which chimeras form a variegated pattern is related to the architecture and developmental program of the plant shoot apical meristem (SAM). Most seed plants exhibit a distinct multi-layered structure in the SAM, with an outer tunica layer and an inner corpus layer. Chimeric plants can be classified as periclinal, mericlinal, or sectorial, based on the color patterns they display, which reflects the cell layer in which the initial albino cell resides and at which stage of SAM development the albino cell first arose [3, 4].

Albino plant cells are defective in some aspects of chloroplast function, the essential photosynthetic organelle that also plays a role in many metabolic pathways [5]. Each cell contains several hundred copies, up to 10 000, of the plastid genome (plastome) [6], each consisting of an approximately 120 ~ 160-kb circular DNA that harbors 120 genes, of which 80–90 encode proteins. Structurally, the plastome comprises a pair of inverted repeats, separated by a long single-copy region and a short single-copy region [7]. In response to endogenous developmental programs and environmental signals, plants differentiate proplastids into several types of mature plastids, including chloroplasts, etioplasts, leucoplasts, amyloplasts, and chromoplasts. Chloroplasts perform photosynthesis, while other plastids contribute to non-photosynthetic reactions for various physiological functions or metabolite biosynthesis [8].

**Figure 1:**
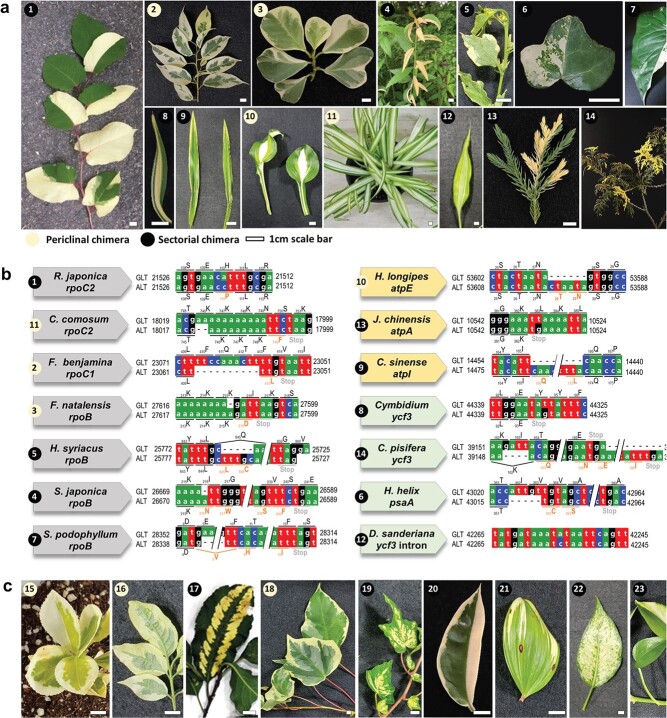
Chimeric plant materials and variation in their plastomes between green and albino sectors. a, Representative chimeric leaves of the 14 plants with plastome polymorphisms identified in this study. 1, *Reynoutria japonica*; 2, *Ficus benjamina*; 3, *Ficus natalensis*; 4, *Spiraea japonica*; 5, *Hibiscus syriacus*; 6, *Hedera helix*; 7, *Syngonium podophyllum*; 8, *Cymbidium hybrid*; 9, *Cymbidium sinense*; 10, *Hosta longipes*; 11, *Chlorophytum comosum*; 12, *Dracaena sanderiana*; 13, *Juniperus chinensis*; 14, *Chamaecyparis pisifera*. b, Local DNA sequence alignment showing the polymorphism and associated amino acid change between green and albino tissues collected from chimeric leaves. PEP-related genes are shown in gray, ATP synthase-related genes in yellow, and photosystem-related genes in green. c, Representative images of the eight chimeric plant species with identical plastid or intronic variation between green and albino leaf tissues. From the left: 15, *Euonymus japonicus*; 16, *Euonymus hamiltonianus*; 17, *E. hamiltonianus* “Snow”; 18, *H. helix* with big leaves; 19, *H. helix* with small leaves; 20, *Hoya carnosa*; 21, *Polygonatum odoratum*; 22, *Aglaonema costatum*; 23, *Epipremnum aureum.*

In land plants, plastids are mainly transmitted to the offspring through maternal inheritance [9]. Most plants carry hundreds of copies of identical plastomes called homoplasmy. Meanwhile, some plants contain two or more types of plastomes, called heteroplasmy, in a cell or an individual [10]. Heteroplasmy arises when plastids from both parental gametes are transmitted to the zygote, resulting in a heterogeneous plastid genotype [9]. During cell division, each plastid randomly segregates into one of the two daughter cells and is either maintained in the same ratio as the mother cell or segregated out, leading to homoplasmic sectors that contain a single plastome [11]. Chimeric plants with normal green alongside abnormal albino leaf tissues within a single individual have been reported in many species, including red pineapple (*Ananas bracteatus*), evening primrose (Genus *Oenothera*), sunflower (*Helianthus annuus*), and ivy (*Hedera* sp.) [11–13, 18]. These phenotypes may have occurred following a mutation in the plastome [11]. Comparison of normal and mutant plastomes revealed various genes conferring mutant phenotypes in *H. annuus* [18] and evening primrose [47]. However, most of the genes responsible for chimerism remain unknown in most ornamental plants.

All plastid genes are transcribed by two RNA polymerases: a plastid-encoded RNA polymerase (PEP) and a nucleus-encoded RNA polymerase (NEP). NEP is a T3/T7 bacteriophage-type monomeric RNA polymerase encoded by the nuclear gene *rpoT* and regulates the transcription of NEP-dependent genes in plastids, including those encoding PEP subunits. PEP is a bacterial-type multimeric RNA polymerase encoded by *rpoA*, *rpoB*, *rpoC1*, and *rpoC2* in the plastome and governs the expression of photosynthesis-related genes. Plastid genes were previously divided into three classes according to their mode of transcription [14], but recently divided as two groups based on global transcriptome analyses of knockout mutants in PEP. One group of genes is transcribed by both NEP and PEP and another is transcribed only by NEP [15]. In previous research, deletion of genes encoding PEP impairs photosynthesis and results in an albino phenotype [16] or the formation of distinct chimeric leaves [17].

In this study, we investigated 23 diverse chimeric plants belonging to different taxa. We identified 14 mutations in plastid genes responsible for albino phenotypes by sequencing green and albino leaf tissues within a single individual. Our high-throughput approach detected point mutations in the plastome, including single nucleotide replacements or small insertions/deletions (InDels) that introduce non-synonymous or nonsense mutations together with frameshifts in heteroplasmic plastids of chimeric plants. This study provides a novel path to unveiling the genetic basis behind chimeric plants.

## Results

### Identification of 14 heteroplasmy plastomes among 23 chimeric plants

We inspected 23 chimeric plants belonging to 21 angiosperms (nine monocots, 12 dicots) and 2 gymnosperms (Table S1, Fig. 1). Seven plants showed a periclinal chimera pattern with a different color between the leaf edge and the inner leaf, like the spider plant (*Chlorophytum comosum*) or weeping fig (*Ficus benjamina*) (Numbers 2, 3, 10, 11, 15, 16, 18 in Fig. 1)*.* The other 16 plants were all sectorial chimeras, with atypical patterns consisting of different ratios of green and albino areas that vary from leaf to leaf, such as with Japanese knotweed (*Reynoutria japonica*) or wax plant (*Hoya carnosa*) (Table 1, Table S1).

**Figure 2:**
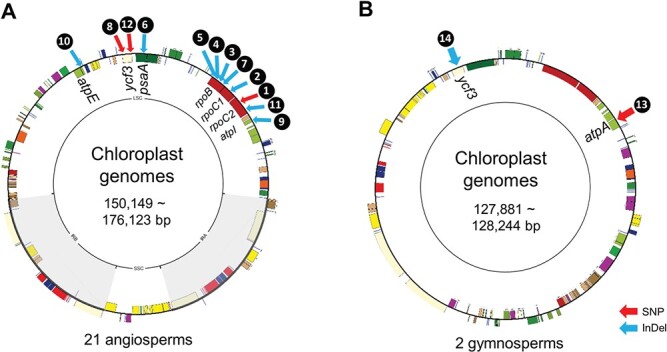
Consensus plastome maps and albino genes in 14 chimeric plants. Each arrow indicates the location of a polymorphism between green and albino plastomes; the numbers refer to the species, as in in Fig. 1a. Red, SNP; blue, InDel. **a,** Complete representative plastome map for the 21 angiosperm species. **b,** Complete representative plastome map of the two gymnosperm species *Chamaecyparis pisifera* and *Juniperus chinensis*.

As the leaf variegation is basically related to the function of the plastid [5, 17, 18], we sequenced separately for genomic DNAs from fresh samples of green leaf tissue (GLT) and albino leaf tissue (ALT) in the chimeric plants. We assembled their plastomes *de novo*, resulting in circular molecules ranging in size from 127 884 to 176 123 bp. The 21 angiosperms plastomes exhibited the typical quadripartite structure consisting of a large single-copy region (LSC), a small single-copy region (SSC), and two inverted repeats (IRa and IRb) (Fig. 2a), while the two gymnosperms plastomes lacked the IR structure (Fig. 2b). Of the 23 chimeric plants, nine plants contain identical homoplasmic plastomes from GLT and ALT; importantly, the remaining 14 chimeric plants were heteroplasmic, as the plastome assembled from green leaf tissues (GLT-PT) differed by single point mutations compared to that assembled from albino leaf tissue (ALT-PT). Nuclear ribosomal DNAs (nrDNA) of each species were also assembled, but no difference was detected between those from GLT and ALT (Table S1).

**Table 1:** Summary of plastid mutations identified in 14 chimeric plants

								**Genotype**		**Depth (Ratio)**			
**#** ^**a**^	**Taxonomy**		**Species**	**Type** ^**c**^	**Gene**	**Mutation**	**Position (bp)**			**Green**		**Albino**	
	**C** ^**b**^	**Family**						**Green**	**Albino**	**Wild**	**Mutant**	**Wild**	**Mutant**
1	AE	Polygonaceae	*Reynoutria japonica*	S	*rpoC2*	SNP	21 519	A	C	668 (96.8%)	22 (3.2%)	0 (0%)	1227 (100%)
2	AE	Moraceae	*Ficus* *benjamina*	P	*rpoC1*	10-bp deletion	23 057	CCAAACTTTT	–	243 (79.2%)	64 (21.8%)	8 (2.6%)	296 (97.4%)
3	AE	Moraceae	*Ficus* *natalensis*	P	*rpoB*	1-bp insertion	27 609	–	A	338 (92.4%)	28 (7.6%)	25 (14.3%)	150 (85.7%)
4	AE	Rosaceae	*Spiraea* *japonica*	S	*rpoB*	1-bp insertion	26 665	–	A	242 (90.0%)	27 (10.0%)	2 (1.4%)	119 (98.35%)
5	AE	Malvaceae	*Hibiscus* *syriacus*	P	*rpoB*	5-bp insertion	25 770	–	GCAAA	72 (60.5%)	47 (39.5%)	34 (17.3%)	162 (82.7%)
6	AE	Araliaceae	*Hedera* *helix*	S	*psaA*	5-bp deletion	43 015	ATTGT	–	136 (wild)^d^ 748 (mutant)^d^			
7	AM	Araceae	*Syngonium podophyllum*	S	*rpoB*	14-bp deletion	28 335	AAAATGAGGGATGAAAATGAGGGATG	–	464 (76.2%)	145 (23.8%)	32 (11.0%)	258 (89.0%)
8	AM	Orchidacea	*Cymbidium hybrid*	S	*ycf3*	SNP	44 331	T	G	299 (100%)	0 (0%)	37 (8.9%)	378 (91.1%)
9	AM	Orchidacea	*Cymbidium sinense*	P	*atpI*	21-bp insertion	14 423	–	CAACCAACTCCAATACTTTA	112 (78.3%)	29 (21.7%)	6 (2.6%)	224 (97.4%)
10	AM	Asparagaceae	*Hosta longipes*	P	*atpE*	6-bp insertion	53 593	–	TATTAG	85 (98.8%)	1 (1.2%)	6 (3.9%)	149 (96.1%)
11	AM	Asparagaceae	*Chlorophytum comosum*	P	*rpoC2*	2-bp deletion	18 005	AA	–	118 (92.9%)	9 (7.1%)	16 (10.1%)	143 (89.9%)
12	AM	Asparagaceae	*Dracaena sanderiana*	S	*ycf3* *intron*	SNP	42 255	A	C	197 (100%)	0 (0%)	14 (6.8%)	192 (93.2%)
13	G	Cupressaceae	*Juniperus chinensis*	S	*atpA*	SNP	10 533	T	G	42 (100%)	0 (0%)	0 (0%)	54 (100%)
14	G	Cupressaceae	*Chamaecyparis pisifera*	S	*ycf3*	4-bp deletion	39 143	GATT	–	45 (97.8%)	1 (2.2%)	0 (0%)	74 (100%)

^a^Serial number for each species.

^b^lades: AE, angiosperms and eudicots; AM, angiosperms and monocots; G, gymnosperms.

^c^lassification of chimera based on leaf pattern. P, periclinal chimera; S, sectorial chimera.

^d^Plastid mutation was identified with the DNA extracted from a whole spotted leaf.

SNP, single nucleotide polymorphism.

**Figure 3:**
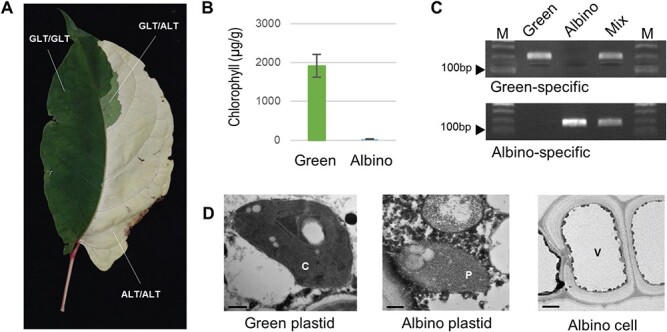
Morphology, chlorophyll contents, and cell structures of the green and albino leaf sectors of *Reynoutria japonica*. a, Representative *R. japonica* leaf showing three types of chimeric tissue: Normal green, pale green, and albino were denoted as GLT/GLT, GLT/ALT, and ALT/ALT for adaxial and abaxial sides, respectively. b, Chlorophyll contents in green and albino leaf sectors. Data are shown as means ± standard deviation from three biological samples. c, Gel electrophoresis of an allele-specific marker to validate the SNP detected in *R. japonica*. GLT, green sector only; ALT, albino sector only; Mix, DNA from mixed leaf tissues containing both green and albino sections. d, Transmission electron microscopy analysis of the plastids in green (GLT) and albino (ALT) leaf sectors. C, chloroplasts in GLT. P, protoplasts in ALT. Albino tissues had abnormal cell structures with a large vacuole (V; right). Scale bars in each picture represent 0.5 μm for GLT, 0.2 μm for ALT, and 2 μm for ALT cells.

These plastid mutations were present across the taxa examined here. Both gymnosperms were heteroplasmic when comparing the plastomes derived from each sector. Of the nine monocot chimeric plants tested, six were heteroplasmic. Likewise, six of the 12 dicot chimeric plants tested were heteroplasmic. Three of the four chimeric plants belonging to the Asparagaceae family were heteroplasmic. However, although the three chimeric common ivy (*Hedera helix*) plants showed different variegated patterns, only one showed a mutation in the albino plastome. We also identified more cases of homoplasmy in pale-yellow leaf sectors or in spotted leaves (Fig. 1c). The leaf chimeric pattern of two *Euonymus* (Numbers 15 and 16 in Fig. 1c) and two *H. helix* (Numbers 17 and 18 in Fig. 1c) species was similar to that seen in the two *Ficus* species (Numbers 2 and 3 in Fig. 1a), but their albino sectors were yellow rather than white in those homoplasmic plants without mutation in the plastome (Numbers 15–17 in Fig. 1c). We also observed yellowish albino sectors in three homoplasmic plants: scented Solomon’s seal (*Polygonatum odoratum*), Devil’s ivy (*Epipremnum aureum*), and lucky bamboo (*Dracaena sanderiana*). Spotted evergreen (*Aglaonema costatum*), Himalayan spindle (*Euonymus hamiltonianus*), and common ivy (*H. helix*) plants were heteroplasmic, and their leaves presented a spotted albino pattern*.*

### Plastid genes responsible to albino phenotype in chimeric plants

We then compared the GLT and ALT plastomes from all plants to inspect stable albino genes across the species as well as mutational hotspots and their role in plastid function. Indeed, we detected 14 independent genic mutations in 14 different chimeric plants affecting eight plastid genes (Table 1 and Fig. 1b). Ten chimeric plants harbor small InDel mutations, resulting in the introduction of early translation termination codons or in the addition/deletion of several amino acids. Four chimeric plants showed single nucleotide polymorphisms (SNPs) that cause single amino acid substitutions in RpoC2 (*R. japonica*), introduce early stop codons in *ycf3* (*Cymbidium* hybrid) or *atpA* (Chinese juniper [*Juniperus chinense*]), or affect an intron in *ycf3* (*D. sanderiana*). We identified seven mutations in genes encoding PEP subunits (*rpoB*, *rpoC1*, and *rpoC2*), four mutations affecting photosystem-related genes (*psaA* and *ycf3*), and three mutations in genes encoding ATP synthase subunits (*atpA*, *atpE*, and *atpI*). For *rpoB* and *ycf3*, which were targeted by multiple mutations, each species harbored a specific mutation not shared with other species carrying a mutation in the same gene.

We next estimated the number of copies for wild-type and mutant plastomes in the sequenced GLT and ALT samples, using the number of reads mapping to each plastome (Table 1). When necessary, we distinguished plastid sequence and mitochondrial plastid DNA (MTPT) based on flanking sequences (Fig. S1). A large portion of the sequencing reads were wild type with a mixture of some mutation type for the GLT plastome. In sharp contrast, almost all of the reads from the ALT plastome shared the mutation, supporting the notion that each mutation disrupts some aspect of chloroplast function, leading to albino sectors or leaves.

### Functional characterization of the *rpoC2* mutant identified in albino leaves of *R. japonica*

The ornamental chimeric plant *R. japonica* produces three types of leaves: entirely green, entirely white, and chimeric (Fig. 1a and Fig. 3a). ALT from *R. japonica* contained just 1.3% of the chlorophyll contents of GLT from the same plant (Fig. 3b). We also established that ALT cell structures are very distinct from those of GLT cells, as determined by transmission electron microscopy (TEM). Indeed, GLT samples contained normal chloroplasts with complete and intact thylakoid structures and typical leaf cell compartments (Fig. 3d). By contrast, ALT samples contained proplastids lacking the typical grana structure or even thylakoid membranes seen in green chloroplasts. In addition, most ALT cells were filled by a large vacuole.

**Figure 4:**
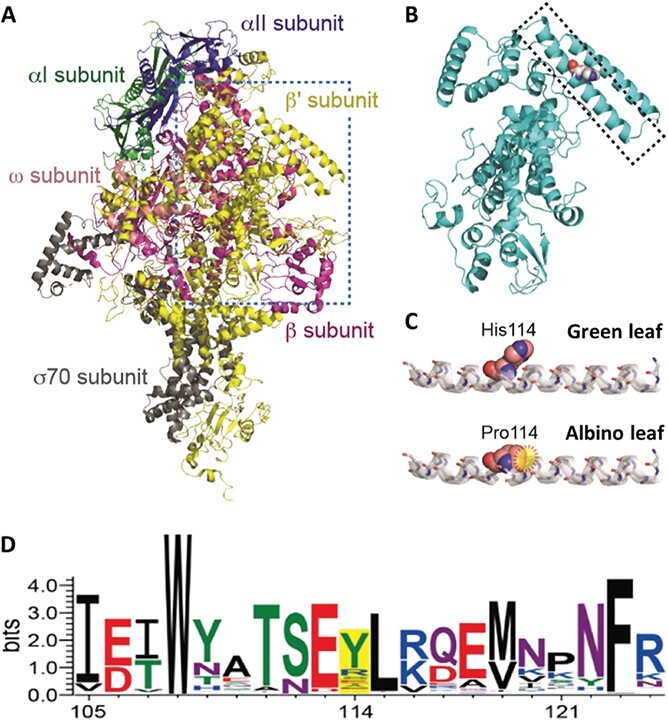
Structural prediction model of the plastid-encoded RNA polymerase of *Reynoutria japonica*. a, Structure of *Thermus thermophilus* RNA polymerase (PDB code: 1IW7), shown as a ribbon diagram. The individual subunits are colored as follows: αI subunit (green), αII subunit (blue), β subunit (magenta), β’ subunit (yellow), ω subunit (salmon), and σ70 subunit (gray). The β’ subunit used for modeling the *R. japonica* RNA polymerase is highlighted by the dashed box. b, Structural model of the β’ subunit of *R. japonica* RNA polymerase, shown as a ribbon diagram. The α-helix harboring the His-to-Pro substitution is shown in a dashed box, while the proline residue is shown as a space-filling model. c, Magnification of the α-helix containing the polymorphism between the green and albino leaf sectors. His114 in green tissues and Pro114 in albino tissues are shown using the space-filling model, and the steric clash between Pro114 and Ala100 in the α-helix is highlighted by a yellow dashed oval. d, Amino acid frequencies of plant RpoC2s near the *R. japonica* His114 residue. The WebLogo was generated using 159 plant RpoC2 proteins retrieved from GenBank. The yellow-shaded region is the His114 residue.

*RpoC2* encodes a critical PEP subunit, which controls the transcription of plastid genes whose encoded proteins form the photosystems [14]. To explore the possible structural consequences associated with the *rpoC2* mutations identified here, we performed a similarity search in the Protein Data Bank (PDB) using RpoC2 as a query, which returned a group of DNA-dependent RNA polymerases (RNAPs). The β’ subunit of RNAP from the bacterium *Thermus thermophilus* (PDB ID: 1IW7, chain D) exhibited the highest alignment score with RpoC2 [19]. *T. thermophilus* RNAP is a multi-subunit complex comprising two α subunits and single β, β’, ω, and σ70 subunits (Fig. 4a). We modeled the structure of RpoC2 and obtained the structural coordinates for Leu11-Thr349 (339 residues) and Ser1125-Leu1317 (193 residues) with a 100% confidence score and 50% sequence identity (Fig. 4b). Superimposition of the modeled RpoC2 structure onto the β’ subunit of *T. thermophilus* RNAP yielded a small root-mean-square deviation (RMSD) of 0.93 Å for 378 Cα atom positions, indicating that *R. japonica* RpoC2 closely resembles the β’ subunit fold of RNAP.

His114, the amino acid affected by the SNP identified between GLT and ALT plastomes, resided in the center of the peripheral helix and appeared to be exposed to solvents (Fig. 4b). Since the peripheral helix is not expected to contact the template DNA or nucleoside triphosphates, the His114Pro substitution would not directly perturb substrate binding interfaces or catalytic sites. We hypothesized, however, that the mutation likely compromises the structural integrity of the helix, which might destabilize the local helical conformation and influence communication between Rpo subunits.

An *in silico* simulation of the change from His114 to Pro114 revealed a large steric clash between the Cδ of Pro114 and the backbone carbonyl oxygen of the nearby Ala110 residue (Fig. 4c). This steric collision may result in general misfolding or in the formation of a helical kink, both of which would be deleterious to proper RNAP activity. Notably, we discovered that proline is never present at the site equivalent to His114 in the peripheral helix, based on an alignment of *R. japonica* RpoC2 and RpoC2 proteins from 159 plant species (Fig. 4d, Table S2). Instead, tyrosine frequently appeared in the position equivalent to His114, suggesting that amino acids with aromatic side chains containing polar functional groups are generally present in this position.

### Transcriptional modification mediated by mutant RpoC2 in *R. japonica*

**Figure 5:**
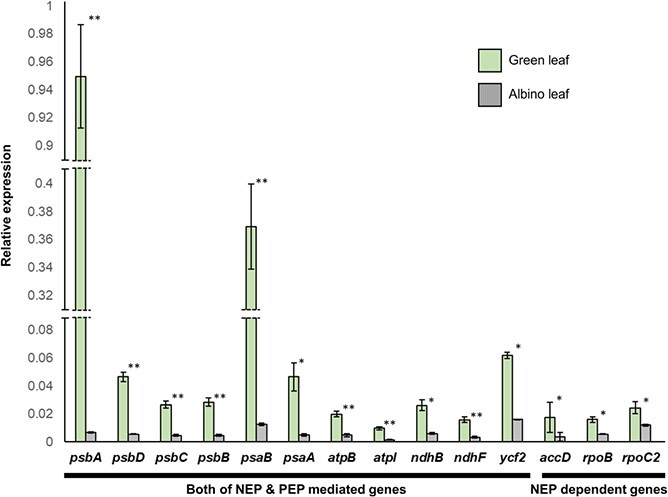
Transcript levels of 14 chloroplast genes in the green and albino leaf tissues in a chimeric *Reynoutria japonica* plant. All RT-qPCR experiments were repeated three times with biological replicates. Data are shown as means ± standard deviation. Asterisks highlight significant differences between the two genotypes, determined by an unpaired Student’s *t*-test (^**^p < 0.01, ^*^p < 0.05).

As RpoC2 is a PEP subunit, any *rpoC2* mutation may alter the transcription of many plastid genes encoding components of photosystems I and II. We therefore conducted RT-qPCR to investigate the transcriptional changes of 14 plastid genes between GLT and ALT. Of these 14 plastid genes, 11 are transcribed by both NEP and PEP, while the remaining three are solely transcribed by NEP (Fig. 5).

The expression levels of all examined plastid genes were much lower in ALT compared to GLT; however, the extent of these differences depended on their functions and the RNA polymerase in charge of their transcription. The transcript levels of photosystem-related genes, which rely on both PEP and NEP for transcription, were 33.3-fold higher in GLT than in ALT, while other genes were 4.81-fold higher between the two tissues (Table S3). PEP- and NEP-dependent genes also showed the most dramatic transcriptional downregulation in ALT; in particular, *psbA* and *psaB* expression decreased 142- and 29-fold, respectively, compared to their levels in GLT (Fig. 5, Table S3). Considering that these genes encode the main photosystem I and II subunits (Yagi & Shiina, 2014), our results suggest that photosystems do not properly form in ALT. The RpoC2 His114Pro substitution in the ALT plastome may therefore lower the transcriptional activity of PEP and impair proper transcription of photosynthetic genes, thereby affecting photosynthesis in albino tissues.

## Discussion

### High-throughput plastome assembly to define heteroplasmy

High-throughput plastome sequencing and assembly have revealed the diversity of the plastome between and within species [20–28]. This diversity provides insights into the evolutionary processes that each plant species has experienced and yields super-barcoding information for each plant species [21, 22, 24, 25, 28–30]. In addition, the diversity of the plastome within species offers diverse tools for the classification of germplasm and the barcoding of breeding lines [20, 22, 27, 28].

In this study, each of plastomes from two leaf sections with contrasting pigmentation (GLT and ALT) was determined in 22 single chimeric individual plants. Heteroplasmic plastomes could also be identified using one whole genome sequence of chimeric plant as in the case of *H. helix* (Table 1). We identified no variation in nrDNA, but detected point mutations in the presumptive causal plastid genes responsible for the chimeric phenotypes in 14 plants; the remaining 9 plants (*P. odoratum*, *E. japonicus*, two individuals of *E. hamiltonianus*, *H. carnosa*, two individuals of *H. helix*, *A. costatum*, and *E. aureum*) showed no polymorphism between their GLT and ALT plastomes (Fig. 1c).

We inspected diverse chimeric plants with different variegation patterns from two species. Of the three *H. helix* individuals sequenced, only one had a polymorphism in its plastome; likewise, the two *E. hamiltonianus* individuals sequenced exhibited opposite chimeric patterns on their leaves but neither had polymorphisms in their plastomes between green and albino sections, suggesting that the chimeric way displayed by a given species does not necessarily correlate with a polymorphism in the plastome. We also hypothesize that susceptibility to mutations causing chimeric leaves differs across taxa and is exceptionally high in the Asparagaceae family. On the other hand, we identified no polymorphisms between GLT and ALT plastomes in Celastraceae chimeric plants. (Table S1).

### Non-lethal preferential albino genes in the plastome of chimeric plants

The transcription of chloroplast genes is controlled by the two RNA polymerases NEP and PEP, both of which are required for the transcription of photosystem genes prior to their assembly into complexes [14, 16]. Here, the sequences of the plastome from green and white leaf sectors or entire leaves from 14 chimeric plants differed by a single nucleotide affecting diverse genes encoding core subunits of PEP or photosystem proteins: *rpoB*, *rpoC1*, *rpoC2*, *psaA*, *ycf3*, *atpA*, *atpE*, and *atpI*. The natural heteroplasmy of *psaA* and *ycf3* were previously reported in albino individual of *Oenothera* [47] and *H. annuus* [18]*.* Although the function of three *rpo* genes have been well studied by reverse genetics, but heteroplasmy of them in natural individuals were rarely reported. The mutations of three ATP synthase genes are firstly reported in this study in terms of albino phenotype.

These unique polymorphisms resulted in the introduction of premature stop codons or in amino acid substitutions (Fig. 1b). Some plastid genes appeared more susceptible to carrying mutations than others, as three PEP genes (out of the four genes in the plastome), three ATP synthase genes (out of six), and one photosystem I gene (out of seven) harbored a potential albino mutation in this study. Not all subunits of the same complex appeared to be the bearer of mutations here, which may indicate that these subunits may perform some other essential function in addition to photosynthesis. Alternatively, increasing the number of sequencing samples for chimeric plants might allow the identification of mutations in more genes than the small list in this study.

Other chimeric plants harbored a mutation in PEP core subunits, which would be expected to abrogate or compromise PEP activity due to early translation termination: This was the case for *C. comosum* (*rpoC2*), *F. benjamina* (*rpoC1*), *F. natalensis*, *H. syriacus*, *S. japonica*, and *Syngonium podophyllum* (*rpoB*) (Fig. 1b). Several studies have shown that mutant plants with defects in *rpo* expression are impaired in chloroplast development [17, 31, 32]. In addition, the identification of PEP-associated proteins and their role in the regulation of PEP activity and chloroplast development further supports the pivotal role of PEP in this process [33–41]. We therefore propose that genetic variation in plastid *rpo* genes results in PEP malfunction, thus causing the dramatic transcriptional decrease in the transcription of photosystem-related genes seen in albino leaves. The absence of photosystems likely reduced the transcription of other genes in albino leaves (Figs. 5 and S2). We detected *ycf3* mutations in three plant species: false cypress (*Chamaecyparis pisifera*), *Cymbidium* hybrid, and *D. sanderiana*. This gene encodes a chloroplast-specific chaperone involved in the assembly of photosystem I (PSI). The *ycf3* mutant of barley (*Hordeum vulgare*) is characterized by a homogeneous light-green leaf pigmentation but is also sensitive to higher temperatures and light due to impaired PSI assembly [42]. However, it is not immediately clear how chimeric leaves arise from the identified polymorphism in *ycf3* in *D. sanderiana*, as the SNP resides in the *ycf3* intron. We hypothesize that this intronic polymorphism affects *ycf3* function via splicing or another uncertain mechanism. However, it is also possible that the chimerism is controlled by nuclear genes, as is likely the case for the other eight homoplasmic plants for which we failed to identify polymorphisms in their plastomes.

Finally, we identified a frameshift in *psaA* in the albino plastome of *H. helix* due to a 5-bp deletion. This gene is a core PSI component; its deletion from the tobacco plastome was associated with severely decreased photosynthetic efficiency, together with downregulation of genes transcribed by PEP [43]. In *J. chinensis* albino plastids, *atpA* harbored a nonsense polymorphism that introduces a premature stop codon. As AtpA is the α subunit of ATP synthase, which is central to ATP production [44], a defect in this protein would negatively affect overall chloroplast energy metabolism [45].

### Albino leaves derived from histidine-to-proline substitution at the α-helix of the RpoC2 subunit in *R. japonica*

The predicted RpoC2 subunit was characterized by a non-synonymous substitution from a histidine to a proline at residue 114 in albino leaves of *R. japonica.* Molecular modeling revealed that this mutation site is located in the middle of the α-helix of the β’ subunit of RNA polymerase. Proline lacks a backbone amide proton that generally serves as a hydrogen-bond donor and thus often breaks α-helical conformations. We speculate that this substitution may affect the helical geometry or stability of the protein, thus inactivating or misfolding RpoC2 (Fig. 4).

Although the *rpoC2* mutation in *R. japonica* does not directly affect NEP, albino leaves also exhibited on average 3.4-fold lower expression levels for genes transcribed only by NEP relative to green leaves. This result might be explained by the differences in light absorption between green and white leaves, as the surface of white leaves will reflect more light away from plant cells. In support of this idea, the transcript levels of *rpoT*, which encodes the single NEP subunit, were reported to increase in proportion with the duration of light exposure received by plants, with an approximately 3-fold increase after a 6-h illumination [46].

### Maintenance and development of albino tissues

Complete albino plants might not survive or be fertile. All chimeric plants carrying polymorphisms in the 14 plastomes are maintained by clonal propagation. Heteroplasmic plant cells possess several hundred plastids of either genotype in a random ratio [9]. Here, we propose a mechanism for the development of albino leaves and the maintenance of their chimeric plants (Fig S2). Spontaneous mutations in plastid genes related to PEP subunits or other photosynthetic genes can be maintained in heteroplasmy form with a normal plastome in GLT without plant lethality. Each plastome randomly segregates between the daughter cells during cell division. Still, after multiple rounds of cell division, some cells may eventually contain only the mutant plastome that carries the albino genotype and evokes the albino phenotype, as suggested by Greiner [9, 11].

The chimeric pattern of each leaf will be decided during development as a function of the cell layer within the SAM where the albino cell(s) arises and spreads [4]. While green leaves might contain a mixture of normal and mutant plastomes, single albino cells will be homoplasmic due to uneven plastid sorting, although albino leaf sections may be either heteroplasmic or homoplasmic depending on the type of chimera. We assumed this hypothesis in this study by determining the number of reads from GLT and ALT mapping to the wild-type or albino plastomes (Table 1). More clearly biased distribution of mutant type plastids is identified in ALT of the sectorial chimera compared to periclinal chimera (Table 1, Fig S3). Weeping fig (*F. benjamina*) and Natal fig (*F. natalensis*) are classified as periclinal chimeras, as the albino pattern appears only at the edge of the leaf, across all leaves. This pattern arises when the entire inner layer of the tunica region in the SAM is albino, while all cells from the outer tunica layer and the inner corpus are wild type. The outer layer mainly contributes to the epidermis, explaining how reads matching the GLT plastome can be detected from albino sections. *R. japonica* is a typical example of sectorial chimera, as the chimeric pattern is different for each leaf and entire albino leaves are common. Since sectorial chimeras are created when albino cells spread to all layers of the SAM, only reads matching the albino plastome will be obtained in ALT.

## Conclusion

Chimeric plants are valuable for ornamental purposes and for understanding the function and biogenesis of plastids such as chloroplasts and leucoplasts. We identified eight plastid genes responsible for albino phenotypes in 14 chimeric plants using high-throughput plastome assembly. Our data suggest that a single mutation in a plastid gene can cause the breakdown of photosystems and prevent chloroplast development in albino leaf tissue. Since chloroplasts are essential for plant survival, mutations in any important chloroplast gene are typically embryo-lethal; therefore, mutant plastomes are usually maintained in chimeric plants in natural conditions rather than as pure albino plants, which would not undergo photosynthesis. Understanding the organellar genomes of these natural or induced chimeric plants will broaden our understanding of plastome dynamics.

## Materials and methods

### Plant materials

Chimeric plants for Japanese knotweed (*R. japonica*), weeping fig (*F. benjamina*), Natal fig (*F. natalensis*), Japanese euonymus (*Euonymus japonicus*), Japanese spiraea (*Spiraea japonica*), and two Himalayan spindle (*E. hamiltonianus*) individuals were provided by Hantaek Botanical Garden, Yongin, Gyeonggi-do, Republic of Korea (http://www.hantaek.co.kr/), Seoul National University Arboretum, Suwon, Republic of Korea (http://arbor.snu.ac.kr/) and Medicinal Plant Garden, College of Pharmacy, Seoul National University, Goyang, Republic of Korea (https://snuherb.snu.ac.kr). Completely green and completely white leaves from a single plant were used for all analyses of chlorophyll contents, cell structures, plastid structures, complete plastome sequences, and chloroplast gene expression (Fig. 1a, 1c, Table S1). The ornamental plants Chinese juniper (*Juniperus chinensis*), false cypress (*C. pisifera*), wax plant (*H. carnosa*), scented Solomon’s seal (*Polygonatum odoratum*), spotted evergreen (*Aglaonema costatum*), Devil’s ivy (*E. aureum*), variegated arrowhead plant (*Syngonium variegata*), plantain lily (*Hosta longipes*), *Cymbidium sinense*, spider plant (*C. comosum*), and lucky bamboo (*Dracaena sanderiana*) were purchased from plant nurseries. A chimeric common ivy (*H. helix*) sample was collected on Jeju Island. A chimeric plant derived from *Cymbidium* hybrid (*Cymbidium sinense* [♀] × *C. goeringii* [♂]) was provided by the Advanced Radiation Technology Institute (Jeongeup, Jeollabuk-do, Korea) [49, 50]. The chimeric cultivar “Purpureus Variegatus” of *Hibicus syriacus* was supplied by the National Institute of Forest Science (Suwon, Korea).

### Measurement of chlorophyll contents and cellular ultrastructures

The chlorophyll contents of the green and white leaves of *R. japonica* were determined spectrophotometrically in three replicates as previously described [51]. Total chlorophyll was extracted from homogenized leaf tissue using 80% (v/v) acetone, and the absorbance of the solution was measured at 647 and 663 nm using a spectrophotometer (Powerwave XS; BioTek, Winooski, VT, USA).

TEM was performed using a previously described method [52] with some modifications. Small pieces of green and white leaves from *R. japonica* were fixed in modified Karnovsky’s fixative (2% [w/v] paraformaldehyde, 2% [w/v] glutaraldehyde, and 50 mM sodium cacodylate buffer, pH 7.2) under low vacuum for 24 h and then washed twice with 50 mM sodium cacodylate buffer, pH 7.2, for 20 min each time. The samples were then processed with 1% (w/v) osmium tetroxide in 50 mM sodium cacodylate buffer, pH 7.2, at 4°C for 2 h and washed twice with distilled water at room temperature. After *en bloc* staining overnight in 0.5% (w/v) uranyl acetate at 4°C, the samples were dehydrated in a graded ethanol series using propylene oxide and then infiltrated with Spurr’s resin and polymerized at 70°C for 24 h. The fixed samples were sectioned to 60 nm using an ultramicrotome (EM UC7; Leica Microsystems, Wetzlar, Germany), mounted onto copper grids, and stained with 2% (w/v) uranyl acetate for 7 min, followed by Reynolds’ lead citrate for 7 min at 25°C. The samples were then visualized using a transmission electron microscope (JEM1010; JEOL, Tokyo, Japan).

### DNA extraction and whole-genome sequencing

The green and white leaves of *R. japonica*, *J. chinensis*, and *C. pisifera* were individually ground in liquid nitrogen using a mortar and pestle. Each region from one leaf was excised with a razor blade and ground independently for plants with green and white sectors within the same leaf (Fig. 1a). Total genomic DNA was extracted using a modified cetyltrimethylammonium bromide (CTAB) protocol [53] or a DNA extraction kit (Exgene Plant SV mini kit, GeneAll Biotechnology, Seoul, Korea) following the manufacturer’s instructions. The quality and concentration of the extracted DNA were determined with a UV spectrophotometer (Nanodrop ND-1000; Thermo Fisher Scientific, Waltham, MA, USA) and by agarose gel electrophoresis. Paired-end DNA sequencing libraries were generated using a TruSeq Nano kit (Illumina, San Diego, CA, USA) and subsequently sequenced by Lab Genomics Inc. (Seongnam, Republic of Korea) using an Illumina MiSeq genome instrument, according to the standard protocol provided by the manufacturer. The sequences and plastid information for the albino leaf tissue (ALT) of *E. hamiltonianus* (Number 17 in Fig. 1) were identical to that of normal tissue from our previous report [30].

### Plastome assembly and annotation

The plastome sequence for each species was assembled by *de novo* assembly of low-coverage whole-genome sequences (dnaLCW) in the CLC Assembly Cell (ver. 4.6 beta; CLC Inc., Aarhus, Denmark) [21, 22] and validated using manual curation of raw read mapping. Raw paired-end reads were trimmed by CLC quality trim software with an offset of quality score value of 33. The trimmed reads were then assembled *de novo* with an overlap distance ranging from 150 to 500 bp and a set window size of 32 for the plastome. The initial contigs were extracted using MUMmer [54] and then arranged and merged into a single draft sequence by comparison to the reference chloroplast sequence from Tartary buckwheat (*Fagopyrum tataricum*; GenBank accession number: NC_027161) or other closely related species with an available sequence in the National Center for Biotechnological Information (NCBI) database. The resulting plastome sequences from green and albino tissues were aligned and compared using MAFFT 7 ^55^ to identify polymorphic sites. Gene annotation was conducted using GeSeq (https://chlorobox.mpimp-golm.mpg.de/geseq.html) [56], and circular maps of the plastome were generated using the OGDRAW program (https://chlorobox.mpimp-golm.mpg.de/OGDraw.html) [57].

### SNP validation using molecular markers

To validate the polymorphism between the plastomes of green and white leaves in *R. japonica*, allele-specific dominant markers were designed, with one primer that includes the SNP at its 3′ end. To improve specificity, two nucleotides before the SNP position were replaced with mismatched nucleotides. The sequences of the left primers are 5′-GGATTCATTTCTTGTCGCATGT-3′ (green leaf specific) and 5′-GGATTCATTTCTTGTCGCATCG-3′ (white leaf-specific). The right primer (5′-AGACTCTGGGTTTCCAGCAA-3′) is common to both sequences. The reaction mixture (25 μL) contained 20 ng of template DNA, 0.4 pmol of each primer. PCR was performed under the following conditions: 7 min at 94°C; 35 cycles of 94°C for 20 s, 65°C for 20 s, and 72°C for 20 s; and a final extension at 72°C for 7 min.

### RNA extraction and cDNA synthesis

Total RNAs were extracted from the green and white leaves of *R. japonica* using the RNeasy Mini kit (Qiagen, Hilden Germany). The quality and concentration of extracted RNAs were determined on a UV spectrophotometer (Nanodrop ND-1000). Gene-specific primers were used for first-strand cDNA synthesis using sequence information for each representative plastid gene (Table S4). The reads from whole-genome sequencing obtained for *R. japonica* were mapped to the Arabidopsis (*Arabidopsis thaliana*) *ACTIN1* sequence (GenBank accession number: NC_003071) to design gene-specific primers for *RjACTIN1* (Table S4). First-strand cDNAs were synthesized using a SMART cDNA Synthesis kit (Takara Bio, Kusatsu, Japan) according to the manufacturer’s protocol.

### Quantitative PCR (qPCR) analysis and structural modeling for RpoC2 mutation

Gene-specific primers were designed for qPCR (Table S5). qPCR was performed on a LC480 LightCycler 480 instrument (Roche, Basel, Switzerland) with a RealHelix Premier qPCR Kit containing SYBR Green with ROX (NanoHelix, Daejeon, Republic of Korea). The qPCR reaction mixture (20 μL) contained 20 ng of template DNA, 0.5 pmol of each primer. The qPCR conditions were as follows: 5 min at 95°C; 45 cycles of 95°C for 20 s, 58°C for 20 s, and 72°C for 20 s; and a final extension at 72°C for 7 min. The relative expression levels were normalized to *RjACTIN1* levels as reference using the ∆∆Ct method of LightCycler 480 Software with default parameter (Roche Diagnostics, ver 1.5.0.39). All qPCR amplifications were repeated three times. A paired *t*-test was used to compare the transcriptional differences of each gene between green and white leaves using Prism (GraphPad, San Diego, CA, USA).

DNA and protein sequences were aligned using the MAFFT program [55]. The alignments were visualized using the WebLogo program [58]. The Phyre2 program was used to model the RpoC2 protein structure [59] based on the *T. thermophilus* RNA polymerase structure (subunit of PDB ID, 1IW7) as a template. The structural models were visualized using the PyMol program (The PyMOL Molecular Graphics System, Version 2.0 Schrödinger, LLC., Cambridge, MA, USA).

## Acknowledgments

We thank Hantaek botanical garden, Seoul National University Arboretum, Medicinal Plant Garden of college of Pharmacy of Seoul National University for kindly provide the chimera materials; Minyoung Lee, Seonheui jeong Yonghyeok Jeong for their help to assembly of the plastomes. This work was supported by the Bio & Medical Technology Development Program of the NRF, MSIP, Republic of Korea (grant no. NRF-2015M3A9A5030733) and grants from the Nuclear R&D Program of the Ministry of Science and ICT (MSIT) and the research program of KAERI, Republic of Korea.

## Author contributions

J.-H.J., H.-S.P., and T.-J.Y. designed the research. T.J.L, J.H.K, H.-Y.K, S.H.K, and J.-B.K provided the chimeric plant samples. J.-H.J., W.C., J.K., Y.S.P, and Y.L. assembled the chloroplast genomes of chimeric plants and conducted comparative genome analysis. J.-H.J. and H.J.K. designed the primers and performed the qRT-PCR. H.J.K. and J.-Y.S. performed the RpoC2 protein modeling. H.-S.P., W.C., and S.-H.K. measured the chlorophyll contents and performed a TEM analysis. H.-S.P., W.C., Y.L., J.-H.J. and T.-J.Y. wrote the manuscript, and H.-S.P., H.J.K., J.-Y.S., J.Y.P., G.J., N.-C.P., and T.-J.Y. revised the manuscript.

## Data availability

**Accession numbers:** All sequence data used in this study have been deposited in the NCBI Nucleotide Database (Table S1). The Arabidopsis *ACTIN1* sequence (NC_003071) and the chloroplast genome sequence of *F. tataricum* (NC_027161) were retrieved from GenBank.

## Conflict interest

The authors declare no competing interests.

## Supplementary data

Supplementary data is available at Horticulture Research online.

## Supplementary Material

Web_Material_uhac246
