# Supplementary material for: High-throughput discovery of plastid genes causing albino phenotypes in ornamental chimeric plants
Source: Hortic Res. 2022 Nov 3;10(1):uhac246. doi: 10.1093/hr/uhac246 (PMC9832966; doi:10.1093/hr/uhac246)
Supplement: Web_Material_uhac246 [file web_material_uhac246.zip › A9. Chimera_manuscript_v17_supporting_v3_HR3.docx]

**Supporting data**

**Sequencing and assembly of the complete plastomes of chimeric plants**

Fresh leaf tissue was collected from green leaf sections (GLT) and albino leaves (ALT) of 23 ornamental plants: *Reynoutria japonica*, *Ficus benjamina*, *F. natalensis*, *Spiraea japonicus*, *Hibiscus syriacus*, *Hedera helix*, *Syngonium podophyllum*, *Cymbidium hybrid*, *Cymbidium sinense*, *Hosta longipes*, *Chlorophytum comosum*, *Dracaena sanderiana*, *Juniperus chinensis*, *Chamaecyparis pisifera*, *Euonymus japonicus*, *E. hamiltonianus*, *E. hamiltonianus* ‘Snow’, *H. helix* with big leaves, *H. helix* with small leaves, *Hoya carnosa*, *Polygonatum odoratum*, *Aglaonema costatum*, and *Epipremnum aureum* (**Fig 1a**)*.* From 0.16 to 2.35 Gb of paired-end reads were generated using the Illumina Miseq platform for GLT and ALT samples, respectively, and were used to assemble the complete plastid genome sequence (PT) of each species for both tissues. For *H. helix* (accession No. 6 in **Table S1**), 3.53 Gb of reads was generated from a single leaf with mixed sectors. Average read coverage for GLT-PT and ALT-PT ranged from 42.3× (GLT-PT of *J. chinensis*) to 1,183× (ALT-PT of *R. japonica*) **(Table S1)**. The assembled contigs were combined to yield the initial circular PT sequences, which were then manually curated to generate the complete PT genome. The PT genomes of five angiosperms had a length of 150,149 to 163,386 bp, with the typical quadripartite structure consisting of a large single-copy region (LSC), a small single-copy region (SSC), and a pair of inverted repeats (IRa and IRb) **(Fig. 2A)**. Angiosperm PT genomes harbored between 106 and 114 genes, including 72 to 80 protein-coding genes, 30 transfer RNAs (tRNAs), and four ribosomal RNA (rRNA) genes. However, those of two gymnosperms, *J. chinensis* and *C. pisifera*, were 127,884 and 128,244 bp long with the non-IR plastid form frequently found in gymnosperm **(Fig. 2b)**. The two PT genomes of gymnosperm each harbor 113 to 115 genes, including 79 to 81 protein-coding genes, 30 encoding transfer RNAs (tRNAs), and four ribosomal RNA (rRNA) genes.

**Characterization of plastid genomes with mutations**

**Plastid genome sequence of *R. japonica***

Genome sequencing was performed on the GLT and ALT samples from a *R. japonica* chimeric plant on the Illumina Miseq platform, resulting in 2.35 and 1.69 Gb of paired-end reads, respectively. The average read coverage for GLT-PT and ALT-PT was 691× and 1,183×, respectively (or an average of 294× and 700× per Gb for GLT-PT and ALT-PT data, respectively) **(Supplementary Table 1)**. Both GLT-PT and ALT-PT were 163,386 bp in length and had a typical quadripartite structure comprising a 87,531-bp large single-copy region, a 13,555-bp small single-copy region, and a pair of 31,150-bp inverted repeats (IRa and IRb) **(Fig. 2a)**. The PT genomes each harbor 114 genes, including 80 protein-coding genes, 30 encoding transfer RNAs (tRNAs), and four ribosomal RNA (rRNA) genes. Both PT genomes were identical except for a single nucleotide polymorphism (SNP), consisting of a T in the GLT-PT sequence and a G in the ALT-PT sequence, in the coding region of *rpoC2*, causing a non-synonymous amino acid change from histidine (AAT) in GLT to proline (AAG) in ALT for residue 114 or RpoC2. The SNP was confirmed by PCR with allele-specific primers **(Fig. 3c)**. An inspection of the sequencing reads overlapping this SNP revealed the heteroplasmy of the GLT sample and the homoplasmy of the ALT sample: The GLT-PT sequence contained 668 reads harboring T and 22 reads with G, while all 1,227 reads from carried a G at this position (**Table 1**).

**Plastid genome sequence of *F. benjamina***

A total of 1.24 and 1.23 Gb of paired-end reads were generated for GLT and ALT samples, respectively, corresponding to average read depths of 346× (GLT) and 359× (ALT). The length of the assembled plastid genomes was 160,329 bp for GLT-PT and 160,319 bp for ALT-PT, due to a 10-bp deletion within *rpoC1* in ALT-PT causing an early termination of translation from the introduced frameshift. The *rpoC1* gene encodes one of the subunits of plastid-encoded RNA polymerase (PEP); such a mutation would be expected to have the same effect as the *rpoC2* mutation from *R. japonica.*

**Plastid genome sequence of *F. natalensis***

A total of 1.18 and 1.26 Gb of paired-end reads were generated for GLT and ALT, respectively, resulting in average read depths of 496× (GLT) and 257× (ALT). The length of the assembled plastid genomes was 160,561 bp for GLT-PT and 160,562 bp for ALT-PT, due to a 1-bp insertion within *rpoB* in ALT-PT causing an early termination of translation due to the introduced frameshift. RpoB is also a PEP subunit, suggesting that a *rpoB* mutation will behave similarly at *rpoC1* or *rpoC2* mutations.

**Plastid genome sequence of *J. chinense***

A total of 1.22 and 1.20 Gb of paired-end reads were generated for GLT and ALT, respectively, for average read depths of 42× (GLT) and 50× (ALT). The length of the assembled plastid genomes was 128,244 bp for both GLT-PT and ALT-PT, with the loss of one IR, which is typical for gymnosperm plastid genomes. A SNP was detected in *atpA* for the ALT-PT genome, resulting in a truncation of the encoded protein due to the introduction of an early translation stop codon. AtpA is the α subunit of ATP synthase, which is involved in ATP production ^44^; a defect in this gene negatively affects overall energy metabolism in chloroplasts ^45^.

**Plastid genome sequence of *C. pisifera***

A total of 0.93 and 1.06 Gb of paired-end reads were generated for GLT and ALT samples, respectively, with average read depths of 49× (GLT) and 66× (ALT). The length of plastid genomes was 127,884 bp for GLT-PT and 127,881 bp for ALT-PT; like *J. chinense*, the *C. pisifera* plastid genome lacks one IR copy. The difference in size is caused by a 4-bp deletion in *ycf3* in ALT-PT, resulting in a frameshift and delayed termination of translation of the Ycf3 protein encoded by ALT-PT. *ycf3* was reported as an essential gene for the assembly of photosystem I ^42^.

**Plastid genome sequence of *Cymbidium***

One *Cymbidium* hybrid and one *Cymbidium sinensis* were sequenced. A total of 2.12 and 2.00 Gb of paired-end reads were generated for GLT and ALT, respectively, from the *Cymbidium* hybrid. The genome length was 150,149 bp for both GLT-PT and ALT-PT. ALT-PT carried a nonsense mutation in *ycf3*. An inspection of sequencing reads overlapping with this SNP indicated the presence of reads of mitochondrial origin in both plastid sequences (**Table 1 and Fig. S3**). In the *Cymbidium* hybrid, the GLT-PT sequence consisted of 299 reads with A at the SNP position, in addition to 60 mitochondrial reads with A. The ALT-PT genome sequence showed a mixture of three types of reads: 37 reads with A (GLT-PT type), 378 reads with C, and 77 mitochondrial reads with A (**Fig. S3**)

In *C. sinensis*, a total of 1.0 and 0.9 Gb of paired-ends reads was generated from GLT and ALT samples, respectively*.* The length of GLT-PT and ALT-PT was 150,149 and 150,173 bp, respectively, due to a 21-bp insertion in the *atpI* coding region in ALT-PT. AtpI also encodes a subunit of ATP synthase. The extension of the protein might affect protein folding or structure and thus impair protein function.

**Plastid genome sequence of *H. helix***

Three *H. helix* chimeric individuals with different leaf morphology were sequenced: One is a sectorial chimera with inconsistently patterned leaves; the other two individuals are typical periclinal chimeras with different leaf sizes (big or small). For the periclinal chimeric plant, genomic DNA was extracted from green and white sectors as one sample and sequenced, generating 3.5 Gb of paired-end reads. The lengths of the GLT-PT and ALT-PT genomes were 156,680 and 156,675 bp, respectively, due to a 5-bp deletion in the *psaA* coding sequence in ALT-PT resulting in early termination of translation. PsaA is an essential protein in the reaction center of photosystem I ^43^; a point mutation in this gene was previously reported in *Helianthus annuus* variegated leaves ^18^*.*

Sequencing of the other *H. helix* chimeric individuals was performed as described for other plants, generating 1.28 and 1.73 Gb of sequence from GLT and ALT (big leaf *H. helix*) and 1.45 and 1.18 Gb of sequence from GLT and ALT (small leaf *H. helix*). We detected no differences between the GLT-PT and ALT-PT sequences from either individual.

**Plastid genome sequence of *H. longipes***

A total of 1.11 and 1.18 Gb paired-end reads were generated for GLT and ALT, respectively, resulting in average read depths of 112× (GLT-PT) and 184× (ALT-PT). The length of the assembled plastid genome was 156,653 bp for GLT-PT and 156,659 bp for ALT-PT, caused by a 6-bp insertion in *atpE* and resulting in an addition of two amino acids. AtpE is a subunit of ATP synthase, together with AtpI and AtpA.

**Plastid genome sequence of *Spiraea japonica*, *Syngonium podophyllum*, and *Hibiscus syriacus***

A total of 0.59 and 0.16 Gb of paired-end reads were generated for GLT and ALT, respectively, from *S. japonica*. The average read depths were 283× (GLT-PT) and 154× (ALT-PT). The lengths of the plastid genomes were 156,137 bp for GLT-PT and 156,136 bp for ALT-PT. In *S. podophyllum*, 1.53 and 1.11 Gb of paired-end reads was generated, resulting in assembled plastid genomes of 163,988 and 163,974 bp for GLT-PT and ALT-PT, respectively. In *Hibiscus syriacus*, 0.87 and 0.81 Gb of paired-end reads were generated from GLT and ALT samples, respectively. The length and average read depth for each plastid genome were 160,892 bp and 115.21× for GLT_PT and 160,897 bp and 245.66× for ALT-PT.

InDels in the *rpoB* coding sequence were detected in ALT-PT in all three chimeric plants: a 1-bp deletion in *S. japonica*, a 14-bp deletion in *S. podophyllum*, and a 5-bp insertion in *H. syriacus*, likely producing nonfunctional truncated proteins as with *F. natalensis.*

**Plastid genome sequence of *C. comosum***

A total of 1.08 and 1.05 Gb of paired-end reads were generated for GLT and ALT, respectively, for average read coverage depths of 143× (GLT-PT) and 220× (ALT-PT). The length of the resulting assembled plastid genomes was 154,337 bp for GLT-PT and 154,336 bp for ALT-PT. A 1-bp deletion in *rpoC2* was observed in ALT-PT, resulting in a truncated RpoC2 and presumably malfunction of PEP, as with *R japonica*, *F. benjamina*, and *F. natalensis*.

**Plastid genome sequence of *D. sanderiana***

A single SNP was found in the *ycf3* intron in *D. sanderiana*, which did not change the protein sequence. The nucleotide sequence around the SNP was ‘AAAT[A]TAA’ from GLT-PT and ‘AAAT[C]TAA’ from ALT-PT (the SNP is shown in brackets). Considering typical donor sequences, the SNP is unlikely to affect splicing directly but may have indirect effects. Alternatively, the causal gene responsible for the albino phenotype may be mitochondrial or nuclear.

**Figures and Tables**

**Fig S1.** Depth of sequencing for reads mapping to the plastid or mitochondrial genomes of green and white *Cymbidium* hybrid leaves*.* Screenshot of read alignments around the SNP in *ycf3*. The reads of plastid origin (PT) are labeled with a green arrow; reads of mitochondrial origin (MT) are indicated by black arrows.

**Fig S2.** **Schematic representation of the transcriptional flow in green chloroplasts and how to induce albino phenotypes.**

**a**, Summary of the formation of chimeric leaves, from genetic mutation to organogenesis. **b**, Transcriptional flow of plastid genes in normal and mutant plastids. Mutations in PEP subunit genes result in low expression of most chloroplast genes mediated by NEP and those of other non-PEP-related genes due to the malfunction of chloroplast components themselves.

**Fig S3.** The ratio of sequence read depth of wild-type and mutant reads in GLT and ALT of sectorial and periclinal chimeric plants. GLT and ALT indicates green and albino tissues, respectively, in each plant.

**Table S1.** Summary of sequencing statistics and plastid information of chimeric plants used in this study. (Excel file)

**Table S2.** Frequency of amino acids at the residue corresponding to *Reynoutria japonica* H114 in 159 plant RpoC2 sequences.

**Table S3.** Relative expression levels of plastid-encoded genes, normalized to *ACTIN1*.

**Table S4.** List of primers for the cDNA synthesis of plastid-encoded and nucleus-encoded *ACTIN1* genes*.*

**Table S5.** List of primers used for qPCR analysis.

**Table S2.** Frequency of amino acids at the residue corresponding to *Reynoutria japonica* H114 in 159 plant RpoC2 sequences.

| **Amino acid** | **Number** | **Percentage** |
| --- | --- | --- |
| Tyrosine (Y) | 93 | 58.5 |
| Arginine (R) | 19 | 11.9 |
| Serine (S) | 10 | 6.3 |
| Glutamic acid (E) | 8 | 5.0 |
| Threonine (T) | 6 | 3.8 |
| Phenylalanine (F) | 5 | 3.1 |
| Alanine (A) | 5 | 3.1 |
| Glutamine (Q) | 4 | 2.5 |
| Asparagine (N) | 3 | 1.9 |
| Isoleucine (I) | 1 | 0.6 |
| Leucine (L) | 1 | 0.6 |
| Methionine (M) | 1 | 0.6 |
| Cysteine (C) | 1 | 0.6 |
| Histidine (H) | 1 | 0.6 |
| Lysine (K) | 1 | 0.6 |
| Valine (V) | 0 | 0 |
| Glycine (G) | 0 | 0 |
| Proline (P) | 0 | 0 |
| Tryptophan (W) | 0 | 0 |
| Aspartic acid (D) | 0 | 0 |
| Total | 159 | 100 |

**Table S3.** Relative expression levels of plastid-encoded genes using *ACTIN1* as a reference.

| **RNA Polymerase** | **Target** | | **Gene**  **mean Cp** | **Reference**  **mean Cp** | **Target/Ref.** | **Fold change**  **(G/W)** |
| --- | --- | --- | --- | --- | --- | --- |
| NEP  and  PEP | *psbA* | G | 20.75 | 34.08 | 10,524.33 | 141.7 |
|  |  | A | 28.63 | 34.80 | 72.38 |  |
|  | *psbD* | G | 25.17 | 34.13 | 501.90 | 8.3 |
|  |  | A | 28.89 | 34.80 | 60.22 |  |
|  | *psbC* | G | 25.97 | 34.13 | 288.67 | 5.7 |
|  |  | A | 29.17 | 34.80 | 50.32 |  |
|  | *psbB* | G | 25.88 | 34.13 | 307.57 | 6.1 |
|  |  | A | 29.16 | 34.80 | 50.45 |  |
|  | *psaB* | G | 22.18 | 34.13 | 3,988.67 | 28.7 |
|  |  | A | 27.69 | 34.80 | 138.67 |  |
|  | *psaA* | G | 25.20 | 34.13 | 501.80 | 9.2 |
|  |  | A | 29.06 | 34.80 | 54.80 |  |
|  | *atpB* | G | 26.38 | 34.13 | 216.33 | 4.1 |
|  |  | A | 29.12 | 34.80 | 52.56 |  |
|  | *atpI* | G | 27.41 | 34.13 | 105.96 | 6.2 |
|  |  | A | 30.72 | 34.80 | 17.06 |  |
|  | *ndhB* | G | 26.00 | 34.13 | 283.20 | 4.3 |
|  |  | A | 28.78 | 34.80 | 65.80 |  |
|  | *ndhF* | G | 26.72 | 34.13 | 171.57 | 4.6 |
|  |  | A | 29.60 | 34.80 | 37.24 |  |
|  | *ycf2* | G | 24.77 | 34.13 | 668.10 | 3.85 |
|  |  | A | 27.39 | 34.80 | 173.40 |  |
| NEP  Only | *accD* | G | 26.59 | 34.13 | 188.23 | 4.6 |
|  |  | A | 29.47 | 34.80 | 40.58 |  |
|  | *rpoB* | G | 26.71 | 34.13 | 173.13 | 2.9 |
|  |  | A | 28.91 | 34.80 | 59.63 |  |
|  | *rpoC2* | G | 26.11 | 34.13 | 264.53 | 2.0 |
|  |  | A | 27.78 | 34.80 | 130.13 |  |

G: Green leaf; A: Albino leaf.

**Table S4.** List of primers for the cDNA synthesis of plastid-encoded and nucleus-encoded *ACTIN1* genes.

| **Gene** | **Primer Sequence (5′****–3′)** |
| --- | --- |
| *psbA* | GGGTAGATCAAGAAAACAGCGG |
| *psbD* | AGCGGTTAGCAGTGACCATT |
| *psbC* | TGCCAGATTCCGCCAAGTAT |
| *psbB* | GGGCGGACACTAAGATGGAAT |
| *psaB* | ATTCCACCGAACGTACTCCC |
| *psaA* | CGCCCAAGAAAGAGACCCAA |
| *atpB* | ACTCCGGATTCTTTCATTTCCA |
| *atpI* | TATCATTCGTAGGTGCGGCT |
| *ndhB* | ATTGATGAGAAGGGGCTGGG |
| *ndhF* | CCAGATCCTAAAAACAACAATGCT |
| *accD* | TTAATTCAAATTGCCTTGCTGTGT |
| *ycf2* | AGAACGAATGGATCTTGTAGGATT |
| *rpoB* | ATCTCTCGTCAAAGGGTGCT |
| *rpoC2* | ACTTGTGTCAGAAATTCCCCGA |
| *ACTIN1* | TCGAACATGATTTGAGTCAT |

**Table S5.** List of primers used for qPCR analysis.

| **Gene** | **Primer sequence (5′–3′)** | |
| --- | --- | --- |
| *psbA* | F | ACAGGAGCTGAATACGCAACA |
|  | R | CTTCATTGCTGCTCCTCCAGT |
| *psbD* | F | TCGAACTTGCCCGATCTGTT |
|  | R | TTGCACCATCACCGTCTTCA |
| *psbC* | F | TGGGCTTTGGCGGGATTTAT |
|  | R | CAATCCACCCTTCTCCCCC |
| *psbB* | F | TTGGGTTTTCTGGCTTGTGC |
|  | R | GAGAGGCTATTCCCCCTGGA |
| *psaB* | F | ATGTCCTGTCCAAGCCAAGG |
|  | R | CCTCATTTTGGTCAACCGGC |
| *psaA* | F | AGCCAGTTTTGGAGCTGCTT |
|  | R | GGCATGTATTTCCACGGTGC |
| *atpB* | F | ACCTACGCCGCCAAATACAG |
|  | R | GGAGCAACTCTCGGACGAAT |
| *atpI* | F | GGTAAAAGAGCCCCTGACCA |
|  | R | TATATCCGGTGTGGAAGTCGG |
| *ndhB* | F | CCTACAGTGATGAATATAAGCGCAA |
|  | R | AGGAATGTTTTTATGCGGTGCT |
| *ndhF* | F | CAGCTCGATAAGACCCCATACC |
|  | R | CCCAATTCCCCCTTCACGTA |
| *accD* | F | AGGGCTTCCAAACACATCGT |
|  | R | GGGCGGCTATACTGATTGGG |
| *ycf2* | F | AATCTAACAGGGGTTCGCGG |
|  | R | TCATCTGCTTCTCACCTTCCG |
| *rpoB* | F | CCTAGCAAACCTCCCGCTAA |
|  | R | AGGTGGTAGGGGTCGAGTTG |
| *rpoC2* | F | TGCACCTTATCCCTTTCCTGC |
|  | R | CGGGTTGGTCCGAGTAGAGA |
| *ACTIN1* | F | GGCCAAAAGGATGCATATGT |
|  | R | TCGAACATGATTTGAGTCAT |

**Fig. S1**


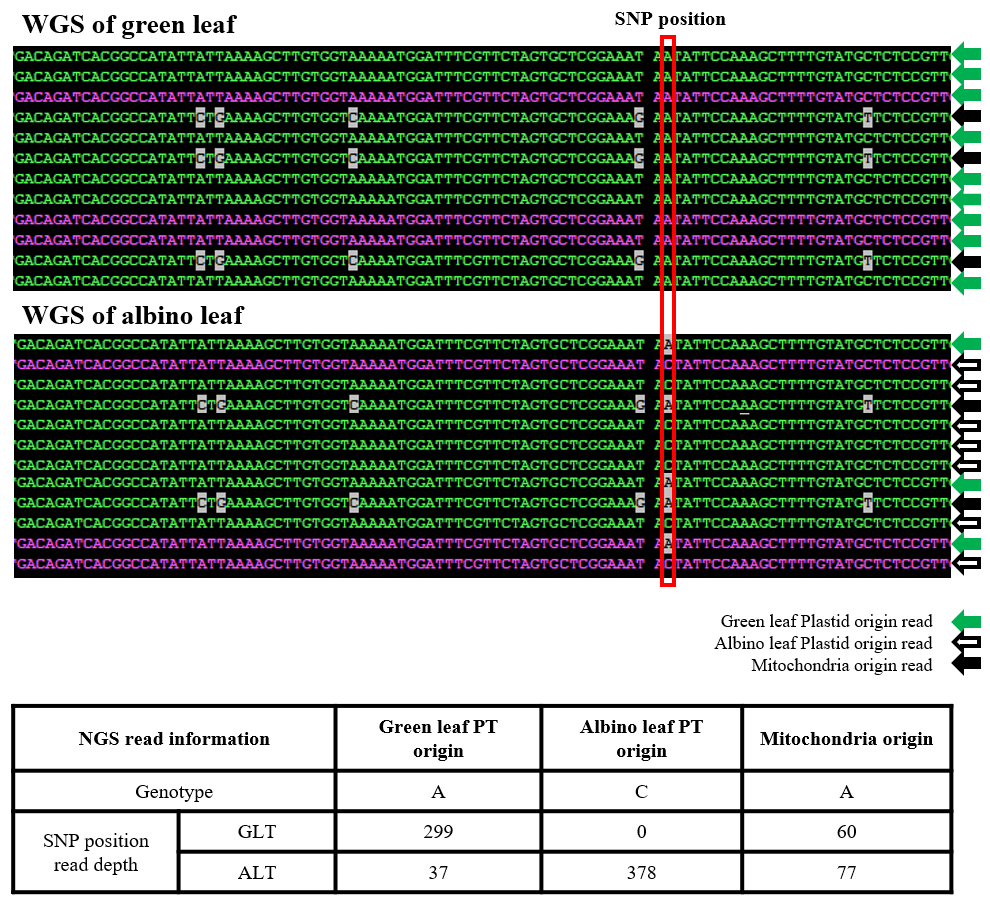


**Fig S2**

**
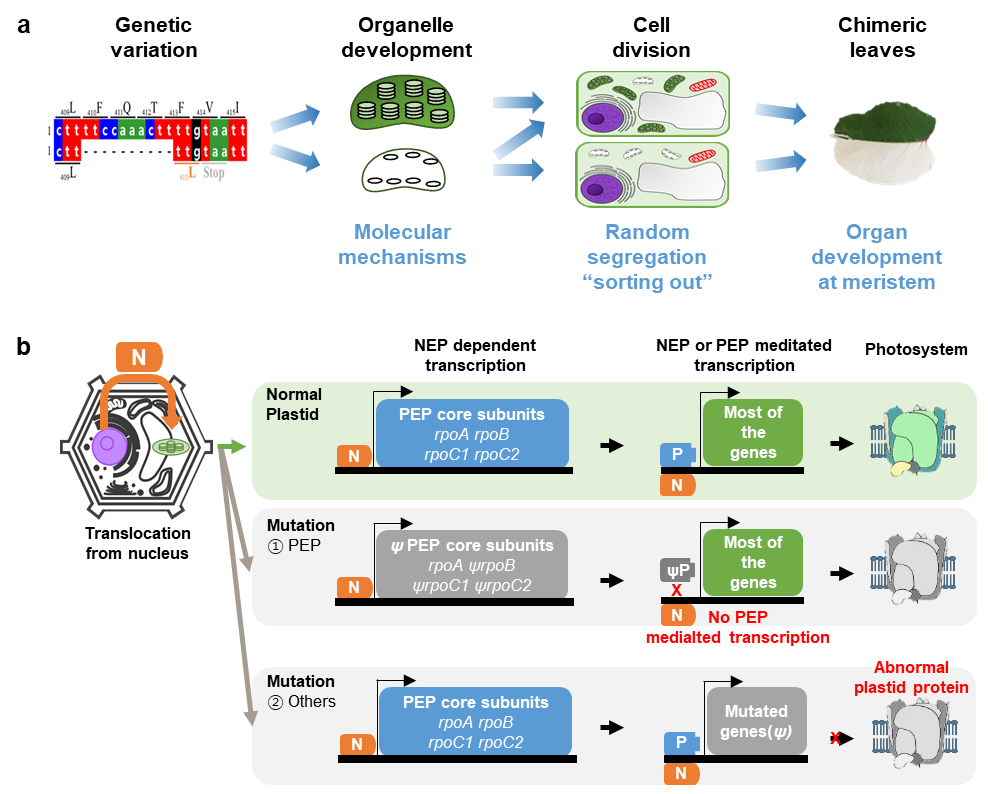
**

**Fig S3.**

**
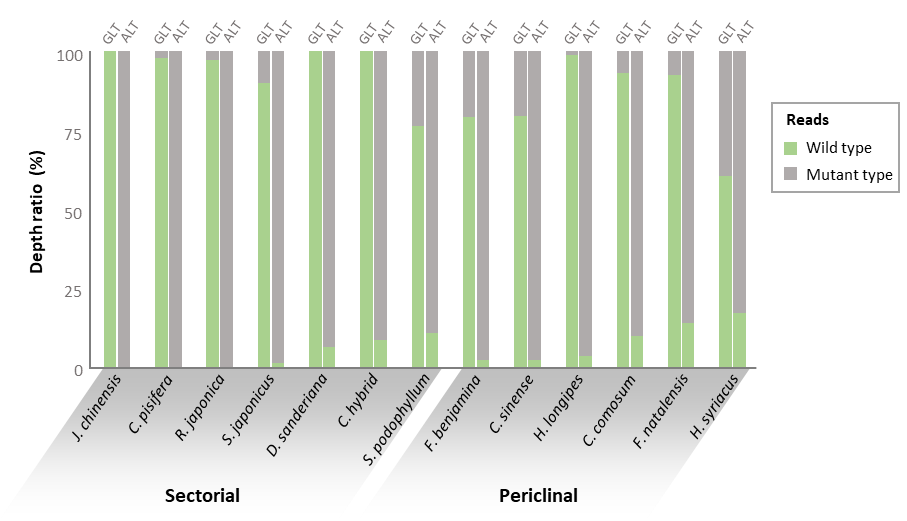
**
